# Exploring cold quarantine to mango fruit against fruit fly using artificial ripening

**DOI:** 10.1038/s41598-019-38521-x

**Published:** 2019-02-13

**Authors:** Abhinandan S. Patil, Dalia Maurer, Oleg Feygenberg, Noam Alkan

**Affiliations:** Department of Postharvest Science of Fresh Produce, Agricultural Research Organization, The Volcani Center, P.O.B 15159, HaMaccabim Road 68, Rishon LeZion, 7505101 Israel

## Abstract

Mango quarantine is based mainly on heat treatment, with a possible deterioration of fruit quality. We studied the effects of cold quarantine (19 days storage at 2 °C) on fruit quality of commercial mango cvs. Keitt and Shelly for three consecutive years. Chilling injury (CI) occurs in mango fruit stored at temperatures lower than 12 °C. By reanalysing our previous transcriptome, we found that under sub-optimal temperature storage (5 °C), the fruit increases its ethylene biosynthesis and osmolarity by activating sugar metabolism, thereby probably reducing its freezing point. Similarly, ripe fruit with higher sugar concentration should be more resistant to cold-storage stress. Here, mango fruit was artificially ripened with 150 ppm ethylene. The control group, stored at 2 °C, suffered from severe CI, whereas the combined treatment of artificial ripening, modified atmosphere (fruit were enclosed in perforated bags) and subsequent low-temperature conditioning resulted in a significant reduction in CI to satisfactory levels for consumer acceptance (taste, aroma and texture). The combined treatment reduced lipid peroxidation and maintained flavour, leading to a novel cold-quarantine treatment for mango fruit. Thus, by reversing the supply chain and storing ripe and ready-to-eat fruit, cold quarantine was enabled for mango, and possibly other chilling-susceptible fruits.

## Introduction

The demand for mango is increasing worldwide^1^. In general, mango is grown in areas with a number of fruit flies. As an example, the Mediterranean fruit fly (*Ceratitis capitata* Wiedemann; Diptera: Tephritidae) requires quarantine measures during export to various countries^2^. Quarantine treatment ensures that specific pests that do not exist in the importing country will not invade via the import of fresh produce^3^. Consequently, mangoes exported to various countries must go through quarantine to ensure fruit fly control. To enable the export of fresh produce, quarantine treatments must eradicate all pests without compromising fruit quality. Since methyl bromide use has been banned in most countries, several post-harvest methods for quarantine treatment have been developed, such as radiation, heat and cold treatments^4,5^. However, these treatments have limitations: heat treatments can impair fruit sensory quality, radiation is relatively expensive and its application is complicated, and cold treatment may cause chilling injuries (CIs). As mango is a tropical fruit, it is very susceptible to cold storage, and cold quarantine has never been considered. Thus, mango quarantine is based mainly on heat treatments^6^.

Cold management of 18 days at 2.2 °C has been accepted by the USDA as a quarantine treatment against fruit fly for many fruit types, including mango^7,8^. The optimum cold-storage temperature for mango fruits is 12 °C, and storage below this temperature can lead to the development of CIs^9^.

Many studies have focused on increasing fruit resistance to sub-optimal temperatures in order to extend fresh produce storage. Modified atmosphere (MA) reduces water loss and significantly reduces chilling in several fruits, including mango^10–12^. Waxing of pomegranate and grapefruit also reduces CI symptoms^13,14^, and in mango, it increases the storage period^15^. Low-temperature conditioning (LTC) reduces external CI in avocado fruit^16,17^ and mango^18^. Interestingly, combined treatments of MA and LTC in cvs. ‘Shelly’ and ‘Keitt’ mango fruit enabled fruit storage at 7 °C with minimal CI (data not shown).

Recently, the mango transcriptome’s response to sub-optimal temperature storage was characterized^9^. Interestingly, one of the main pathways that were elevated was sugar metabolism, where starch is metabolized to mono-saccharides and di-saccharides. The elevated sugar content^9^ probably increases osmolarity and reduces the fruit’s freezing point. Similarly, mango fruit harvested during late season and thus having a minor increased in sugar content (osmolarity) can be stored at lower temperatures, 10 °C instead of 12 °C^19^.

Hence, in the current work, we integrated artificial ripening (AR) with LTC and MA to enhance mango fruit’s resistance to cold. Remarkably, these combined treatments allowed the storage of mango fruit under cold quarantine of 2 °C for 19 days with the minimal development of CIs. Hence, AR combined with other treatments that are known to reduce CI enables the possibility of storing mango fruit at a temperature lower than 12 °C. By reversing the storage paradigm, cold-sensitive fruit can undergo cold-quarantine treatment during export of ready-to-eat fruit.

## Results

### Progression in Ripening Parameters during Cold Quarantine

**Figure 1:**
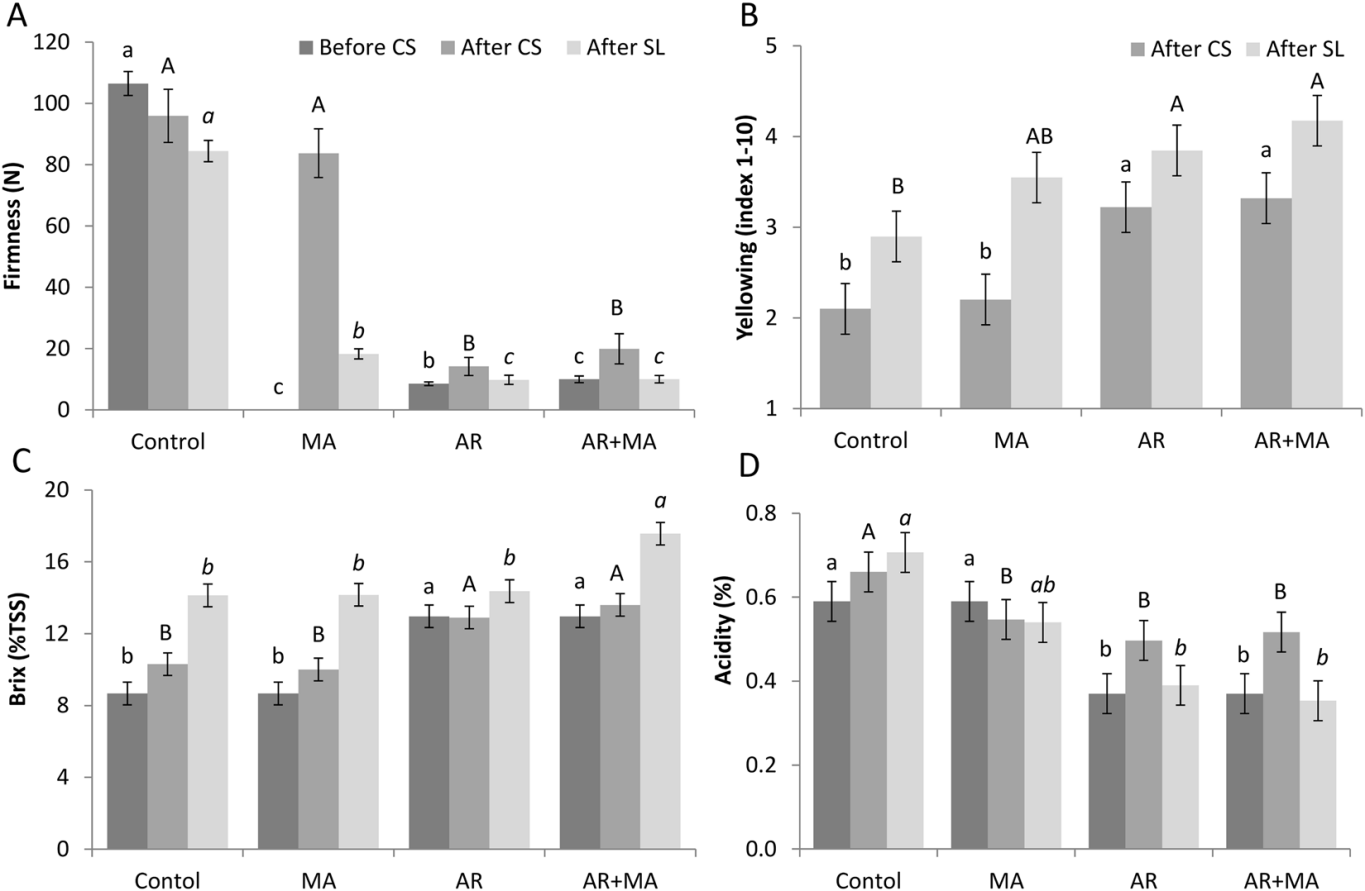
Ripening parameters for ‘Keitt’ mango fruit (2017). Treated (modified atmosphere [MA], artificial ripening [AR] at 150 ppm for 24 hours and the combination of AR + MA) or untreated control mango fruit before cold storage (CS), after CS at 2 °C for 19 days and after a further 4 days of shelf-life at 20 °C (SL) storage were measured for ripening parameters. (**A**) Firmness presented in Newton (N). (**B**) Yellowing (index 1–10). (**C**) Brix (%TSS). (**D**) Acid (% acetic acid). Presented are average values ± SE. Different letters indicating a significant difference at *P* < 0.05.

Our previous results showed that to cope with storage at sub-optimal temperature, mango fruit activates sugar metabolism and degrades starch to mono-saccharides, thereby elevating osmolarity and reducing its freezing point^9^. In a similar manner, during fruit ripening, fruit degrade starch to mono-saccharides. Thus, in the current study, we artificially ripened fruits of mango cv. Keitt and Shelly and integrated the ripening with a MA and LTC. Several experiments were conducted on cv. Keitt during the years 2015 and 2017 and on cv. Shelly in 2016, with similar results. As expected, AR led to close to full ripening of the fruit with significant softening, change in peel colour, reduced acidity and increased Brix (Fig. 1, Table 1). Brix is commonly used for rough measurements of soluble sugars. Indeed, as the ‘Keitt’ fruit ripened in the AR + MA treatment, the Brix (% total soluble solids [TSS]) increased significantly in comparison to the control, from 8.67 to 12.97% (Fig. 1C). After ripening, the elevated levels of Brix (%TSS) in the AR treatment remained nearly constant whereas the fruit in the control group slowly accumulated sugars during storage (Fig. 1C). The increased fruit ripening in the AR + MA treatment was visible relative to controls as the firmness levels dropped swiftly from 106.5 to 10.0 N (before cold storage), whereas in the controls, the firmness decreased gradually, with the fruit remaining firm and not softening well during storage (Fig. 1A). Moreover, fruit treated with AR + MA had reduced acidity relative to controls, which supports fruit ripening (Fig. 1D). Similar results of increased ripening parameters (increased Brix, reduced acidity and firmness) after AR treatment were observed for the two cultivars (Keitt and Shelly) in different years (2015 and 2016, respectively) (Table 1).

**Table 1:** Effect of cold quarantine on post-harvest traits of ‘Keitt’ (2015) and ‘Shelly’ (2016) mango fruit.

Cultivar/Year	Traits	Treatments						
		Harvest	After cold storage			After shelf life		
		Control	Control	AR	AR + MA	Control	AR	AR + MA
Keitt-2015	Brix (%TSS)	7.41 ± 0.50	9.57 ± 0.60^b^	10.98 ± 0.60^ab^	11.38 ± 0.60^a^	12.88 ± 0.50^a^	12.86 ± 0.50^a^	12.90 ± 0.50^a^
	Acidity (%)	1.70 ± 0.10	0.90 ± 0.10^a^	0.70 ± 0.10^b^	0.76 ± 0.10^ab^	0.75 ± 0.10^a^	0.75 ± 0.10^a^	0.70 ± 0.10^a^
	Firmness (N)	—	72.06 ± 2.60^a^	4.28 ± 2.60^b^	5.83 ± 2.60^b^	19.52 ± 2.30^a^	3.85 ± 2.30^b^	5.39 ± 2.30^b^
	Pitting (index 1–10)	—	7.10 ± 0.70^a^	3.92 ± 0.70^b^	1.67 ± 0.70^c^	6.70 ± 0.70^a^	4.91 ± 0.70^ab^	3.35 ± 0.70^b^
	Black spots (index 1–10)	—	4.97 ± 0.50^a^	1.55 ± 0.50^b^	0.55 ± 0.50^b^	6.55 ± 0.50^a^	3.52 ± 0.50^b^	1.62 ± 0.50^b^
	Total decay incidence (%)	—	—	—	—	—	—	2.8 ± 2.8
Shelly-2016	Brix (%TSS)	6.88 ± 0.41	9.37 ± 0.46^b^	13.20 ± 0.46^a^	14.00 ± 0.46^a^	10.82 ± 0.46^b^	13.22 ± 0.46^a^	13.80 ± 0.46^a^
	Acidity (%)	1.06 ± 0.05	1.03 ± 0.05^a^	0.87 ± 0.05^b^	0.85 ± 0.05^b^	0.96 ± 0.05^a^	0.76 ± 0.05^b^	0.76 ± 0.05^b^
	Firmness (N)	88.36 ± 3.32	81.91 ± 3.71^a^	5.63 ± 3.71^b^	4.67 ± 3.71^b^	75.91 ± 3.71^a^	4.90 ± 3.71^b^	3.65 ± 3.71^c^
	Firmness (index 1–10)	10 ± 0.19	10 ± 0.19^a^	3.77 ± 0.19^b^	3.29 ± 0.19^b^	10.00 ± 0.19^a^	3.66 ± 0.19^b^	3.29 ± 0.19^b^
	Colour (Hue)	119.49 ± 1.43	118.31 ± 1.60^a^	96.17 ± 1.60^b^	92.46 ± 1.60^b^	109.46 ± 1.60^a^	79.21 ± 1.60^c^	86.82 ± 1.60^b^
	Yellowing (index 1–10)	10 ± 0.19	1.00 ± 0.12^b^	7.92 ± 0.12^a^	8.05 ± 0.12^a^	1.56 ± 0.12^c^	9.11 ± 0.12^a^	8.63 ± 0.12^b^
	Pitting (index 0–10)	—	9.14 ± 0.26^a^	0.30 ± 0.26^b^	0.00 ± 0.26^b^	10.00 ± 0.26^a^	2.42 ± 0.26^b^	0.00 ± 0.26^c^
	Black spot (index 0–10)	—	6.04 ± 0.40^a^	1.08 ± 0.40^b^	1.15 ± 0.40^b^	9.86 ± 0.40^a^	4.44 ± 0.40^b^	1.24 ± 0.40^c^
	Side decay severity (index 0–10)	—	—	—	—	7.38 ± 0.26^a^	0.86 ± 0.26^b^	0.18 ± 0.26^b^
	Side decay incidence (%)	—	—	—	—	100.00 ± 9.07^a^	30.56 ± 9.07^b^	17.50 ± 9.06^b^

Different superscript letters in a row for each time point represent significant difference at *P* < 0.05.

After cold storage (2 °C for 19 days), after shelf life (20 °C for 4 days), AR, artificial ripening at 150 ppm for 24 hours; MA, modified atmosphere. Different superscript letters (a,b,c) in a row for each time point represent significant difference at *P* < 0.05.

Further, AR + MA treatment ripened the fruit and the colour changed from green to orange as recorded for cv. Shelly in 2016, with hue number decreasing from 118.31 in controls to 92.46 after cold storage, and after shelf life, from 109.46 to 86.82 (Table 1), and yellowing index (1–10) decreasing from 1–1.5 (green) in the control to 8–8.6 (yellow) in the AR + MA treatment after cold storage and shelf life, respectively (Table 1). A similar but less pronounced increase in the yellowing index was observed for cv. Keitt (Fig. 1B).

### Resistance to CI during Cold Quarantine

**Figure 2:**
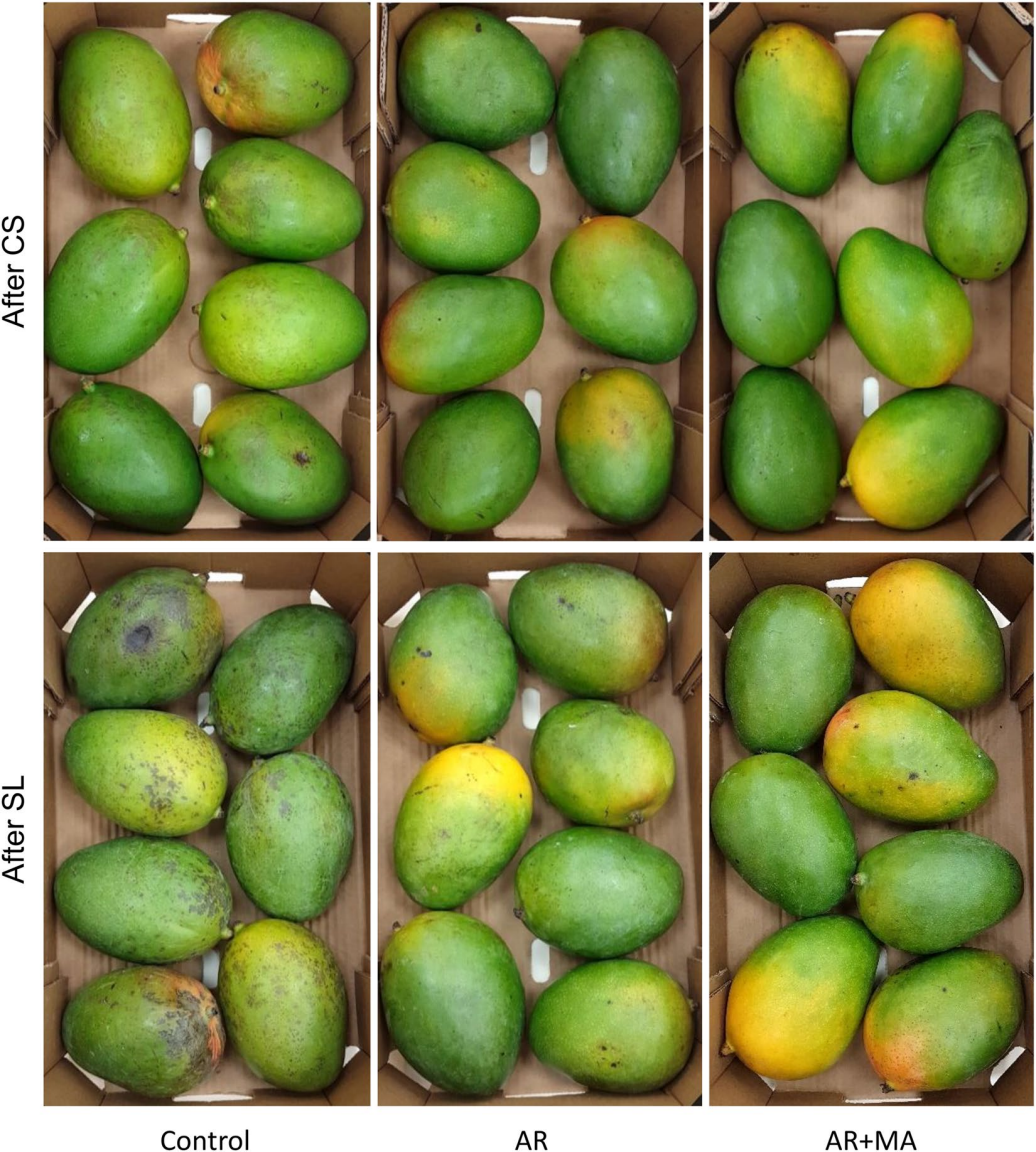
Representative pictures of boxes of cv. ‘Keitt’ mango fruit after cold storage (CS) (upper panel) and after an additional 4 days of shelf life (SL) (lower panel) of untreated controls, fruit treated with artificial ripening (AR) at 150 ppm for 24 hours and those treated with a combination of artificial ripening and modified atmosphere (AR + MA).

**Figure 3:**
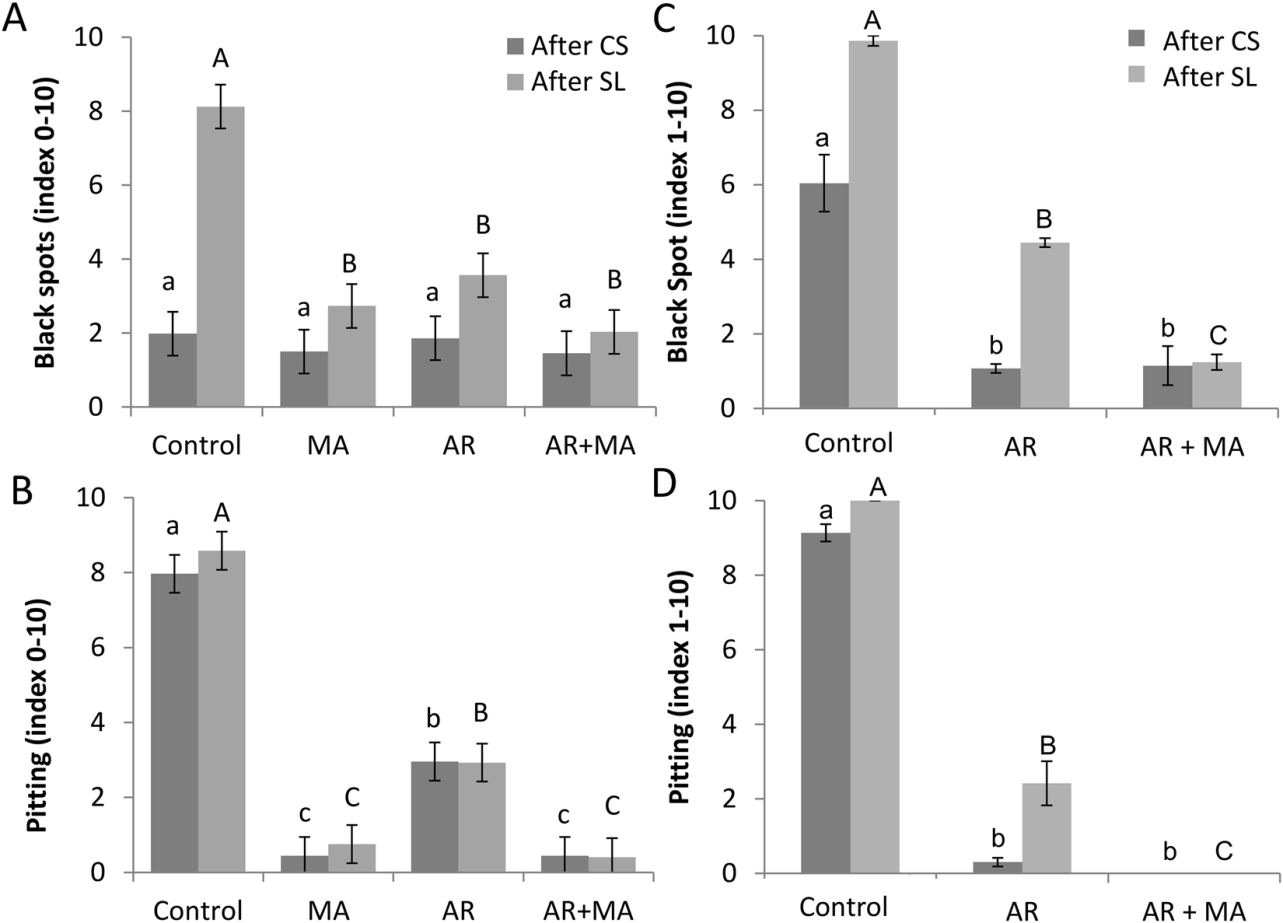
Chilling injury (CI) parameters after cold quarantine of cvs. ‘Keitt’ (2017) and ‘Shelly’ (2016) mango fruit. Treated or untreated mango fruit after cold storage (CS) at 2 °C for 19 days and a further 4 days of shelf-life at 20 °C (SL) storage were measured for CI severity in control, modified atmosphere (MA), artificial ripening (AR) at 150 ppm for 24 hours and AR + MA groups. (**A**) Black spots (index 0–10) and (**B**) Pitting (index 0–10) of ‘Keitt’ (2017) mango fruit. (**C**) Black spots (index 0–10) and (**D**) Pitting (index 0–10) of ‘Shelly’ (2016) mango fruit. Presented are average and SE. Different letters indicate a significant variance of *P* < 0.05.

Cold quarantine requires 18 days of storage at 2.2 °C to eradicate the Mediterranean fruit fly from various fruit types^7^. In the current experiment, we stored the fruit at 2 °C for 19 days, followed by another 4 days of shelf-life storage at 20 °C. CI was expressed and measured in terms of black spots and pitting on the peel. The unripe fruit from the control group suffered from severe CI after cold storage, which further increased after shelf-life storage in both cvs. ‘Keitt’ and ‘Shelly’ (Figs 2 and 3, Table 1, Supplementary Fig. S1). Black spots and pitting were significantly decreased when treated by AR + MA in comparison to the control after storage (Fig. 3, Table 1). For example, black spots (index 0–10) were lowered from very severe (8.2 or 9.9) in the control to mild (2 or 1.2) and acceptable in the AR + MA treatment after shelf-life storage (Fig. 3A,C). Pitting (index 0–10), which is a deterioration of the black spots, was very mild (0.4 or 0) in AR + MA compared to very severe (8.6 or 10) in the control group after shelf life (Fig. 3B,D). These results highlight the importance of AR + MA for maintaining mango quality with close to no CI during cold quarantine. Similar results of reduced black peel spots and pitting were recorded in ‘Keitt’ and ‘Shelly’ (Table 1). Indeed, representative pictures of treated and untreated fruits of ‘Keitt’ (Fig. 2) and ‘Shelly’ (Supplementary Fig. S1) after cold quarantine and an additional 4 days of shelf-life storage clearly show a significant reduction in CI on the AR + MA fruit compared to the untreated fruit, resulting in good-quality ready-to-eat fruit after cold storage.

### Post-harvest Decay during Cold Quarantine

After cold storage, the AR + MA-treated fruit was ready to eat and no decay was observed. However, the minor decay of 2.8% and 17.50% was observed after the additional 4 days of storage at 20 °C in the combined AR + MA treatment for cvs. Keitt and Shelly, respectively (Table 1). In contrast, in the control of Shelly cultivar had 100% of very severe side decay (Table 1). Stem-end rot incidence and severity were quite minor after 4 days of shelf-life storage for all treatments (data not shown). Indeed, representative pictures of treated and untreated ‘Keitt’ and ‘Shelly’ mango fruit after cold quarantine and an additional 4 days of shelf-life storage clearly show a significant reduction in decay in the ripe fruit compared to the untreated fruit (Fig. 2 and Supplementary Fig. S1), with the ready-to-eat fruit remaining in good condition for up to 4 or 5 days. However, it is worth mentioning that after 7 days of shelf-life storage of ready-to-eat fruit, all treatments showed severe decay (data not shown).

### Lipid Peroxidation during Cold Quarantine

**Figure 4:**
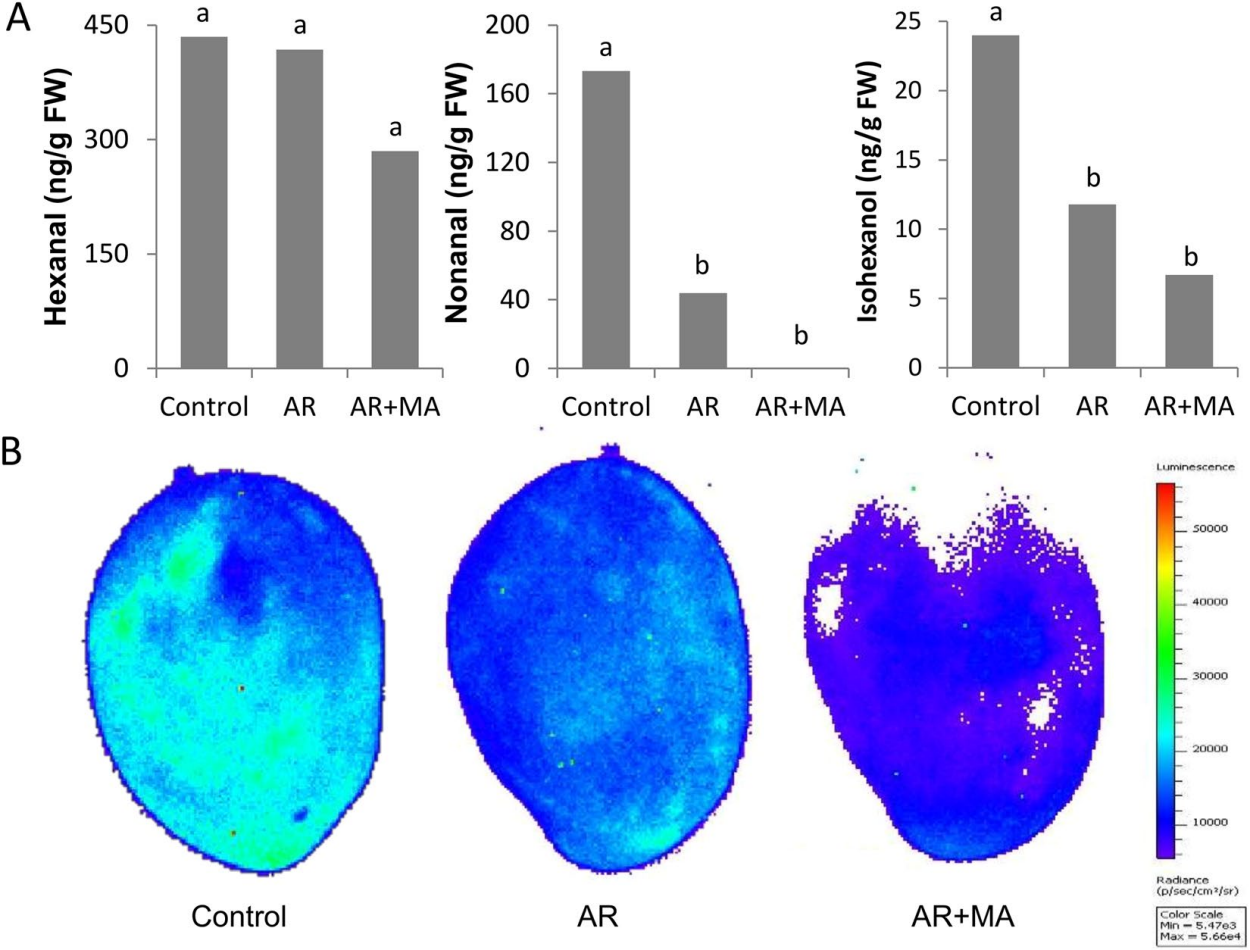
Lipid peroxidation, luminescence and volatiles after cold quarantine of cv. ‘Keitt’ mango fruit and before shelf life. (**A**) Production of volatiles hexanal, nonanal and isohexanol after cold quarantine in treated and untreated mango peel. (**B**) Luminescence over 650 nm for control fruit stored at 2 °C for 4 days (left picture) or fruit stored at 2 °C for 19 days after artificial ripening (AR) at 150 ppm for 24 hours (middle picture) and modified atmosphere combined with AR (right panel). Presented are average values ± SE. Different letters indicate a significant difference at *P* < 0.05.

Luminescence of mango fruit from the control group was high 4 days after storage at 2 °C (Fig. 4B), whereas a significant reduction was recorded after 19 days of cold storage (Supplementary Fig. S2). However, fruit from AR treatment showed lower lipid peroxidation and luminescence after 4 days of storage at 2 °C, whereas those from the combined AR + MA treatment had very low lipid peroxidation after 4 days and after 19 days of storage at 2 °C (Fig. 4B; Supplementary Fig. S2), which indicate a lower CIs in the AR + MA treatments after cold-quarantine.

Lipid peroxidation could also generate secondary compounds, including the volatile compounds, which could cause off-flavors in fruits. GC-MS analysis of lipid peroxidation related volatiles showed that hexanal, nonanol and isohexanol, which are the linolenic acid degradation product, were accumulated by 2.27 and up to 20-fold in the fruit peel of the control compared to the combined treatment. Additionally, ethanol and acetaldehyde was significantly induced in the controlled fruit in comparison to the combined treatments (data not shown).

### Effects of Cold Quarantine on Aroma Volatiles and Acceptance

**Figure 5:**
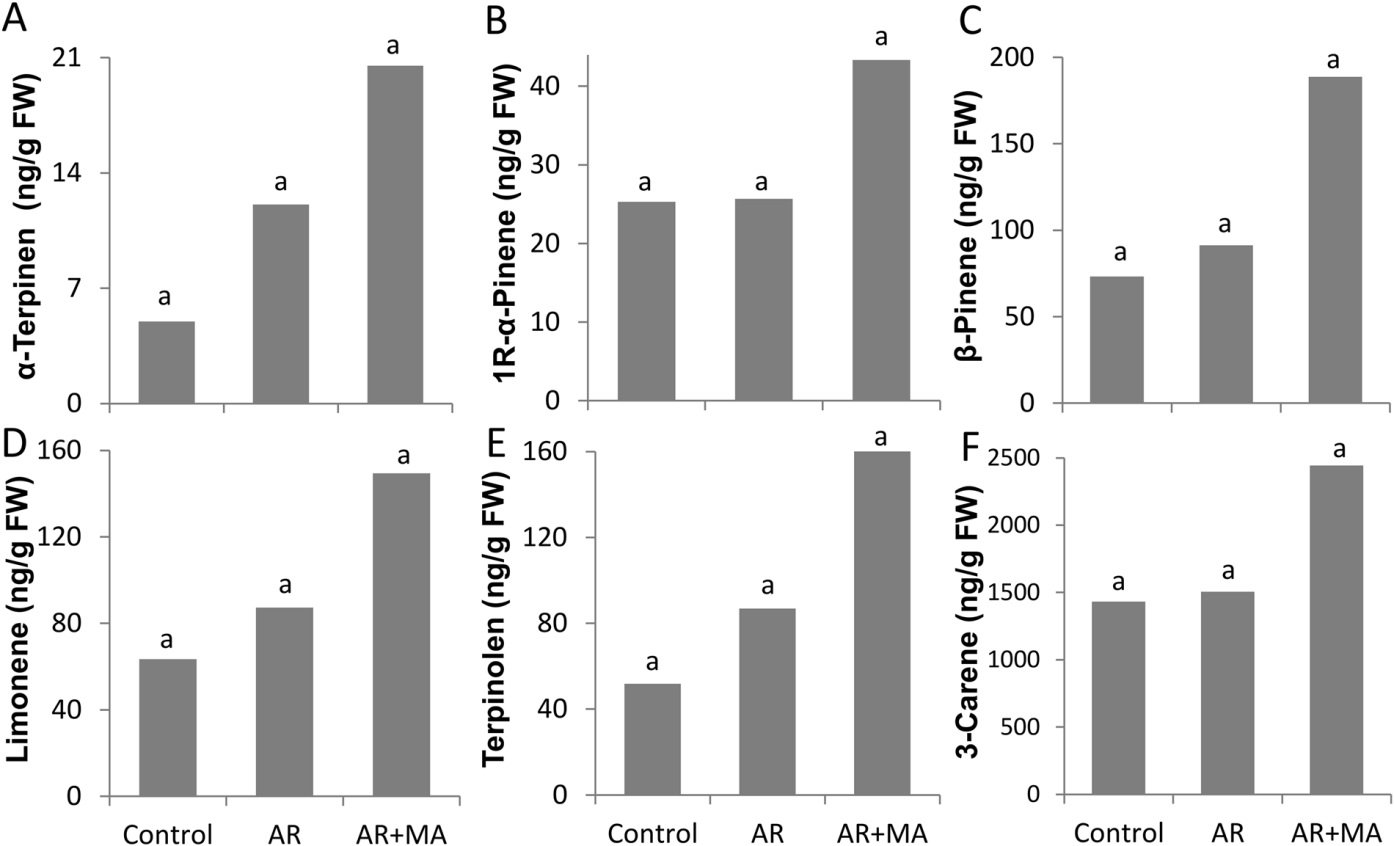
Aroma volatile compounds released after cold quarantine and before shelf life from ‘Keitt’ mango fruits peel. (**A**) α-Terpinen. (**B**) 1R-α-Pinene. (**C**) β-Pinene. (**D**) Limonene. (**E**) Terpinolen. (**F**) 3-Carene. Presented are average values ± SE. Different letters indicating a significant difference at *P* < 0.05.

**Figure 6:**
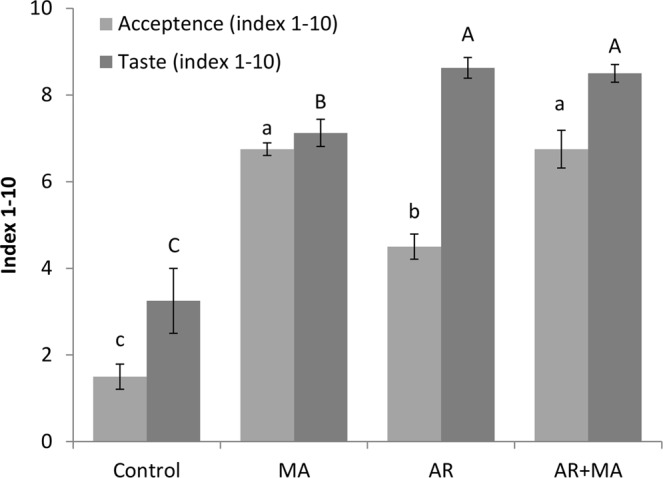
Organoleptic characteristics of cv. ‘Keitt’ mango fruit (acceptance and taste) after cold quarantine and 4 days of shelf life, presented by index 1–10. Presented are average values ± SE. Different letters indicating a significant difference at *P* < 0.05. MA, modified atmosphere; AR, artificial ripening.

Fruit volatiles were characterized in the peel of control fruit and in the AR and AR + MA treatments (Supplementary Info S1). Interestingly, several volatiles that are known to contribute to the general aroma of mango fruit were induced in the AR + MA treatment in comparison to controls: *α*-Terpinen (4.0-fold), 1R-*α*-Pinene (1.7-fold), *β*-Pinene (2.6-fold), Limonene (2.4-fold), Terpinolen (3.3-fold) and 3-Carene (1.7-fold) (Fig. 5), which supported better acceptance and taste among the sensory panel (Fig. 6).

During fruit ripening, acids are degraded and sugars increase, leading to a higher sugar-to-acid ratio—the key parameter determining acceptance and taste among consumers. In our experiment, AR + MA had 4.50- and 2.60-fold higher acceptance and taste index values compared to controls, respectively, after cold quarantine (Fig. 6). Thus, fruit treated with AR + MA showed good quality, taste and aroma, while presenting resistance to the cold-quarantine treatment.

## Discussion

Quarantine management must eradicate all fruit fly stages (eggs, larvae and mature insects). Cold quarantine against fruit fly for various fruit types, including mango, consists of storage at 2.2 °C for 18 days^8^. To develop cold quarantine for cold-susceptible fruit, it is mandatory to reduce CI or induce cold-resistance. Thus, cold quarantine for citrus, bell pepper, pomegranate and avocado includes treatments that reduce CI and induce fruit resistance to cold, such as an initial heat treatment, LTC or a combination of jasmonic acid, MA and conditioning^17,20–22^. However, as a tropical fruit, mango is one of the most susceptible commodities to chilling^23,24^. Mango fruit is commercially stored at 12 °C, and storage below this temperature leads to CI^9^. Therefore, cold quarantine at 2 °C has never been considered for this fruit, and its quarantine is based mainly on heat treatments and to a minor extent on radiation^6^. This study challenges the main paradigm of storing unripe fruit in the cold by proposing storage of ripe fruit, which is much more resistant to cold. Previous unpublished results showed that a combination of treatments, such as jasmonic acid, MA and LTC, which allowed cold quarantine for avocado fruit^17^, reduced CI in unripe mango and enabled fruit storage at 7–8 °C, whereas storage at the lower temperature required for cold quarantine led to substantial CI (data not shown).

**Figure 7:**
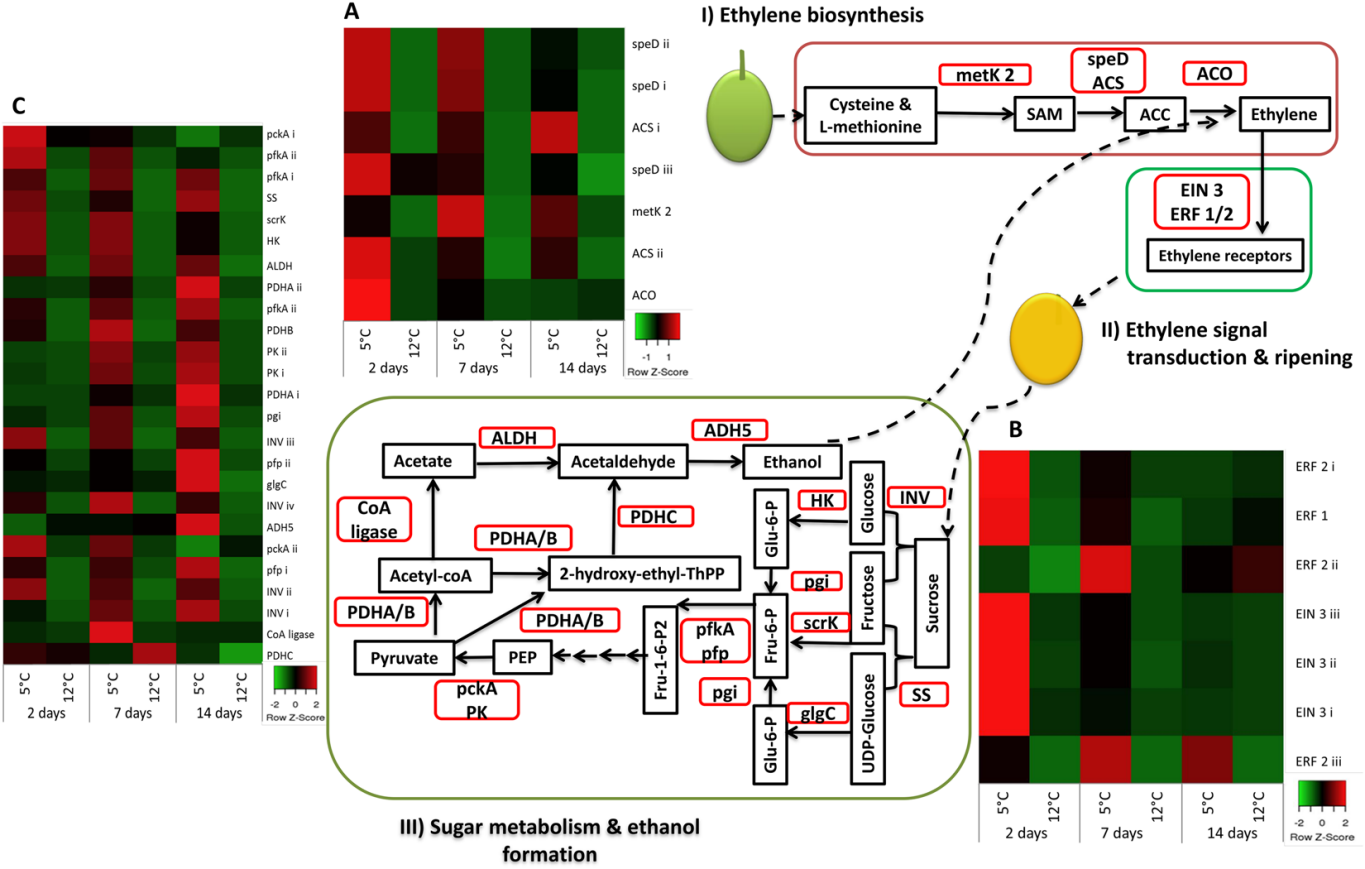
‘Keitt’ mango fruit transcriptomic response to sub-optimal storage associated with ethylene and sugar metabolism. Mango fruit chilling triggers ethylene biosynthesis and signal transduction, which leads to fruit ripening, sugar metabolism and ethanol formation. Transcripts in red rectangles were significantly up-regulated during storage at 5 °C compared to 12 °C. Heat maps for relative expression represent: (**A**) ethylene biosynthesis, (**B**) ethylene signal transduction and ripening and (**C**) sugar metabolism and ethanol formation. Transcript abbreviations and expression values are presented in Supplementary Info S2. Note: Transcriptomic responses studied at 5 °C compared to 12 °C, and not at 2 °C compared to 12 °C as done for cold-quarantine.

In climacteric fruit, including mango, sugar metabolism is coordinated by ethylene, which triggers the onset of ripening^25^. In the present study, exogenous application of ethylene triggered fruit ripening, which was accompanied with elevated brix, reduced acidity, fruit softening and change of color. By re-analysing our ‘Keitt’ mango fruit transcriptome in response to storage at optimal temperature at 12 °C and sub-optimal at 5 °C^9^, and not as was performed at this experiment were the fruit were stored at 2 °C, we found that mango fruit significantly activate the ethylene biosynthesis and signaling in response to sub-optimal storage at 5 °C temperature. Seven transcripts for ethylene biosynthesis were activated: s-adenosylmethionine synthase 2 (metK 2), three isoforms of s-adenosylmethionine synthase (speD), two isoforms of s-adenosylmethionine decarboxylase (ACS), and ACC oxidase (ACO) (Fig. 7A and Supplementary Info S2), along with seven transcripts for ethylene signal transduction: three isoforms of EIN 3 (i–iii), ERF 1 and three isoforms of ERF 2 (i–iii) (Fig. 7B, Supplementary Info S2). The synthesis and signal transduction of ethylene are known to activate fruit ripening and sugar metabolism^26^. Indeed, by re-analysing our previous RNA-seq of mango fruit response to storage at sub-optimal temperature, the pathway of sugar metabolism was strongly activated, including 25 transcripts that led to the degradation of starch to mono- and di-saccharides and ethanol formation (Fig. 7C, Supplementary Info S2). Thus, in response to sub-optimal storage at 5 °C temperature mango fruit activated, ethylene biosynthesis and signal transduction and sugar metabolism (Fig. 7). We hypothesize that these transcripts are triggered in response to fruit storage at a sub-optimal temperature to better cope with cold stress.

Our previous study showed that in mango fruit stored at sub-optimal temperature, starch is degraded to soluble sugars^9^. Degradation of starch to mono- and di-saccharides such as sucrose, fructose, and glucose increases the osmolarity, and these compounds act as cryo-protectants to reduce the freezing point^27^. Indeed, mango fruit that is harvested during the late season have increased TSS (osmolarity) and can be stored at lower temperatures^19^.

In the current study, mango fruit was artificially ripened before storage. Remarkably, ripe fruit (TTS over 13%) were much more resistant to cold storage and had less CI when stored at 2 °C (Figs 2, 3, Table 1 and Supplementary Fig. S1). To eliminate the minor CI that remained after the ripening (AR) treatment, we integrated it with MA and LTC, which are known to reduce CI in various post-harvest commodities^17,22^. MA leads to a reduction of O_2_ and elevation of CO_2_, thus inhibiting respiration leads to increase in mango fruit tolerance to chilling^10^. Thus, ripening by ethylene followed by reduction of respiration by MA resulted in a further reduction in CI to a level that was acceptable to consumers (Figs 2, 6, Table 1 and Supplementary Fig. S1).

CI is known to be strongly correlated with elevated production of reactive oxygen species^28^, which lead to the activation of linolenic acid pathway, lipid peroxidation and degradation^9,29^. The detection of fruit luminescence by *in-vivo* imaging systems can non-destructively pinpoint early lipid peroxidation^9^. In the present study, the integrated treatments of AR + MA had lower luminance, indicating reduced lipid peroxidation (Fig. 4) and CI. This lower lipid peroxidation was also expressed in reduced degradation of linolenic acid and reduced oxylipin C6 and C9 volatiles such as hexanal, isohexanol and nonanol (Fig. 4), which are associated with linolenic acid pathway, lipid peroxidation and degradation in response to chilling stress in mango fruit^9,29^. Thus, this combination of treatments opens the way for cold-quarantine application with cold-susceptible fruit such as mango.

Apart from reducing fruit CI, the most critical aspect of mango fruit storage is retention of fruit flavour, aroma and taste. While some reports have suggested that long heat-quarantine applications can reduce fruit quality^6,30–32^, the cold-quarantine application does not. Indeed, GC–MS analysis of the known mango aroma volatiles α-Terpinen, 1R-α-Pinene, β-Pinene, Terpinolen and 3-Carene^29,33^ after cold quarantine of AM + MA-treated fruit showed their increase in the peel (Fig. 5). Those results were correlated to the results that cold quarantine of AM + MA-treated fruit showed reduced CI, good pulp taste and increased aroma volatiles in the peel, leading to high levels of consumer acceptance. The results presented here show that after cold storage, the fruit was ready to eat. These ready-to-eat fruit could be held at 20 °C for up to 4 days with the maintenance of relatively good quality and low decay.

## Conclusions

Quarantine is vital for global trade^3^. Cold quarantine is easy to apply as it is performed during export shipment. Cold quarantine is applied mainly to chilling-resistant fruits. In this manuscript, we show that by inverting the cold chain and storing ripe and ready-to-eat fruit together with MA and LTC - chilling-susceptible as tropical fruit can be subjected to cold-quarantine treatment while maintaining their fruit quality.

## Materials and Methods

### Fruit Materials

Mid-season mature, full-size and unripe mango fruit (*Mangifera indica* L.) were harvested in August of 2015 and 2017 (cv. Keitt) and August 2016 (cv. Shelly), and transported (1 h) from the Mor storage facility to the Agricultural Research Organization, Volcani Center, Israel. Export-class ‘Keitt’ and ‘Shelly’ fruit weighed between 390 g and 450 g, with 9 ‘Keitt’ fruit and 10 ‘Shelly’ fruit per cardboard box in the cold-quarantine experiment. Fruit was stored at 2 °C for 19 days, with or without the post-harvest treatments detailed below.

### Post-harvest and Cold-quarantine Treatments

To reduce CI and maintain mango fruit quality, the fruit were treated with (1) AR – harvested fruit were artificially ripened with 150 ppm ethylene for 24 hours followed by 2 days of storage at 18 °C; (2) MA – fruit were enclosed in low-density-perforated (30 holes of 0.5 mm) polyethylene bags (StePac, Israel), removed from the bag after 1 day to avoid condensation (97–98% relative humidity [RH], 4–7% CO_2_ and 8–16% O_2_). Apart from the controls, all treatments included (3) LTC – the temperature for both AR and AR + MA treatments was gradually reduced over 3 days: day 1 at 12 °C (RH 74.80%), day 2 at 5 °C (RH 86.90%) and day 3 at 2 °C (RH 91.80%). The controls were not treated and were stored at a 2 °C for 19 days (fruit core temperature of 2 ± 0.25 °C). Cold storage was followed by 4 days of shelf-life storage at 20 °C (RH 63.3%).

Five cardboard boxes, containing 9 fruits (‘Keitt’) or 10 fruits (‘Shelly’), were used for each treatment replication. The room temperature was monitored by DAQ tool -double strand wire logger/data acquisition control system (T.M.I Barak Ltd., Israel). Fruits core temperature was monitored using a MicroLite data Logger LITE5032P-EXT-A (Fourier technologies, Israel) by inserting its probe 3 cm deep, near to the mango stone.

### Physiological Measurements

CI symptoms of mango fruits were examined after cold-quarantine (2 °C for 19 days) and 4 days shelf-life (20 °C) storage. CI severity in terms of black peel spots and pitting were evaluated by CI index (on a scale of 0–10; 0 = no CI, 10 = severe CI). Other physiological parameters were rated on relative scales: colour (scale of 1–10; 1 = green, 10 = orange); fruit firmness (scale of 1–10; 1 = soft, 10 = firm); decay severity (scale of 0–10; 0 = no decay, 10 = severe decay), and decay incidence presented as percentage of decayed fruit in a box.

Other post-harvest parameters were measured using instruments. Firmness was tested via the peel using an 11-mm probe penetrometer (LT-Lutron FG-20 kg, Indonesia) at two points on each fruit (10 fruit per treatment). Fruit colour (hue) was measured by chromometer (Minolta LR-400/410) at two points on each fruit (10 fruit per treatment). TSS was measured by a digital refractometer (ATAGO PR-1, Japan) and presented in percent Brix (%TSS). Acidity concentration was measured by automatic titrator (Metrohm, 719 S Titrino, Switzerland) and calculated based on percent citric acid.

### RNA-Seq Analysis

RNA-Seq of Sivankalyani *et al*., (2016) was re-analysed. Briefly, samples were extracted from mango fruit peel, from fruit that was stored for 2, 7 and 12 days at sub-optimal temperature of 5 °C and optimal (12 °C) temperatures (and not at 2 °C temperature as quarantine requires). Library construction and RNA-Seq analysis were carried out as described previously^9^. Up-regulated transcripts of clusters 2, 4 and 5 were annotated with Kyoto Encyclopedia of Genes and Genomes (KEGG)^34^ and analysed for biosynthesis pathways and signal transduction. Heat maps were prepared for up-regulated transcripts with Heatmapper^35^.

### Identification and Quantification of Volatiles

Aroma and lipid-peroxidation volatiles were identified and quantified by gas chromatograph 7890 A and mass chromatograph 5975 C (Agilent Technologies Inc., USA). After cold-quarantine treatment, mango peel samples (1 g) were collected randomly from five fruit of each replicate in three biological repeats for cv. Keitt in 2017. Samples were immediately stored with 2 mL NaCl (20% w/v) to stop further enzymatic activity in 20-mL dark-coloured glass bottles (LaPhaPack, Germany) with a tight seal. S-2-octanol (Sigma-Aldrich) was used as the internal standard. In each run, NaCl without sample was used as a control. Analytical conditions for the GC–MS run were adjusted as described by^29^. Volatile identification was based on NIST mass spectral database version 5. Identified volatiles were quantified based on internal standard linear retention indices and expressed as µg/kg FW.

### Fruit Organoleptic Characterization

To determine the sensory attributes of ‘Keitt’ mango fruit after cold quarantine, untrained tasting panel of 15 people evaluated the acceptance and taste using an index of 1–10. These sensory attributes were judged for control, MA, AR and AR + MA fruit based on ranked general remarks on impression, sweetness, sourness and off flavours.

### Lipid Peroxidation and Luminescence

A pre-clinical *in-vivo* imaging system (Perkin Elmer, USA) and a highly sensitive charge-coupled camera (CCD) were used to detect alterations in the cellular membrane caused by lipid peroxidation. Fruits of cv. Keitt after cold quarantine was re-acclimatized for 2 h in the dark prior to *in-vivo* imaging system evaluation. Oxidative degradation of lipids was recorded at 640–770 nm wavelength emitted for 20 min as proposed by^29,36^. Data were recorded for three biological replications, and one representative picture is presented.

### Statistical Analysis

Data from three different experiments are presented as average ± SE. One-way ANOVA was used to compare means of treatments to controls with JMP Pro 13.0 statistics software and data were subjected to Duncan’s multiple-range tests. Differences at *P* < 0.05 were considered significant. Indices for black spot (scale 0–10), pitting (scale 0–10), acceptance (scale 1–10), taste (scale 1–10), firmness (scale 1–10), yellowing (scale 1–10) and side-decay severity (scale 0–10) were calculated with the formula:

$${Index}=\sum \frac{{Respective}\,\mathrm{scale}\,\times {Number}\,{of}\,{fruit}\,{present}\,{in}\,{that}\,{level}}{{Total}\,{number}\,{of}\,{fruit}\,{in}\,{the}\,{treatment}}$$

## Supplementary information

Supplementary Fig and Info
