# Supplementary material for: Exploring cold quarantine to mango fruit against fruit fly using artificial ripening
Source: Sci Rep. 2019 Feb 13;9:1948. doi: 10.1038/s41598-019-38521-x (PMC6374360; doi:10.1038/s41598-019-38521-x)
Supplement: Supplementary file 1 — Supplementary Fig and Info [file 41598_2019_38521_MOESM1_ESM.pdf]

Exploring cold quarantine to mango fruit against fruit fly using artificial ripening

Abhinandan S. Patil*, Dalia Maurer*, Oleg Feygenberg & Noam Alkan

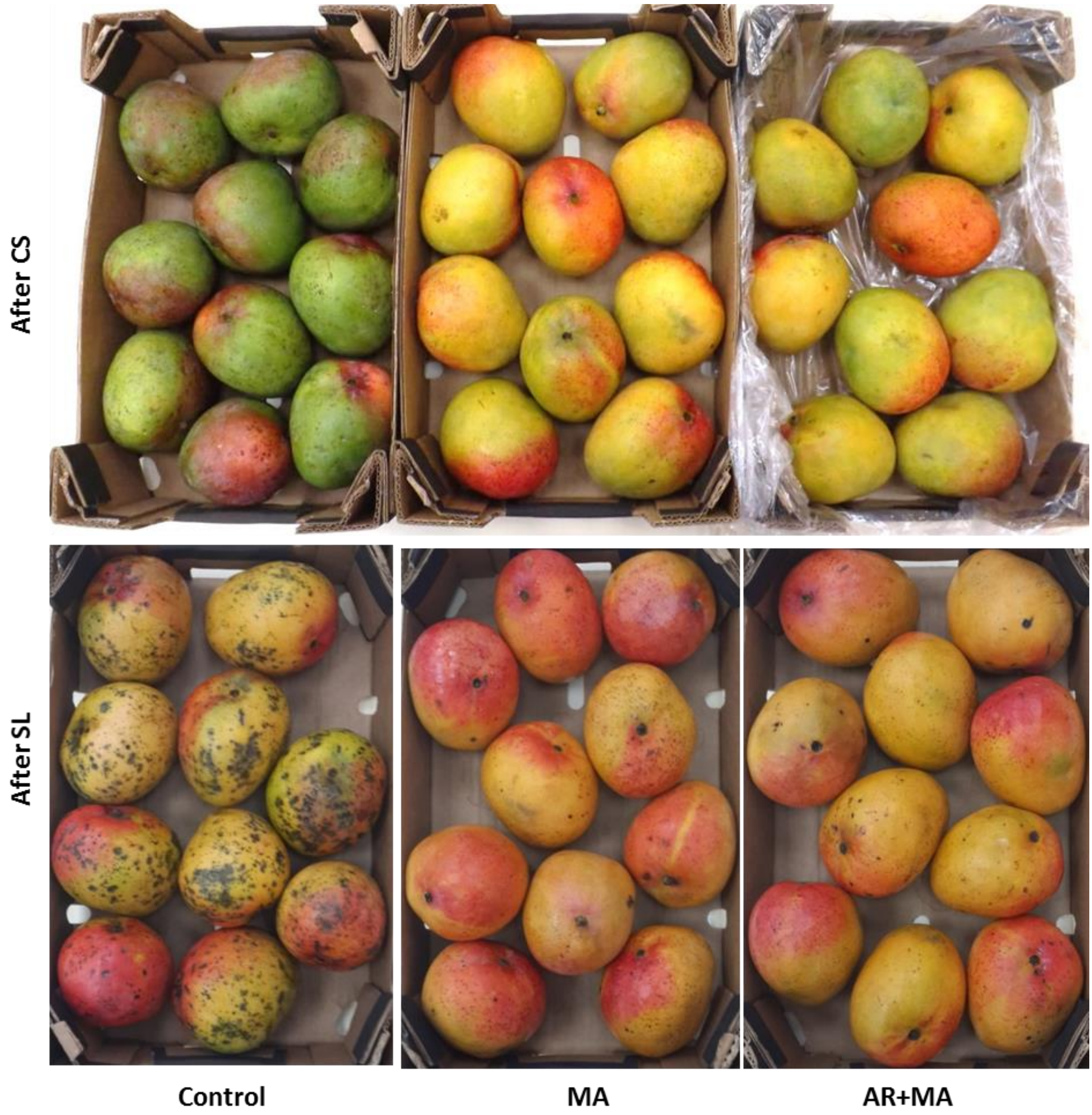

Supplementary Fig. S1. Representative pictures of boxes of 'Shelly' mango fruit after cold storage (CS) (upper panel) and an additional 4 days of shelf life (SL) (lower panel) for control, modified atmosphere (MA) and the combination of artificial ripening and modified atmosphere (AR+MA).

Exploring cold quarantine to mango fruit against fruit fly using artificial ripening

Abhinandan S. Patil*, Dalia Maurer*, Oleg Feygenberg & Noam Alkan

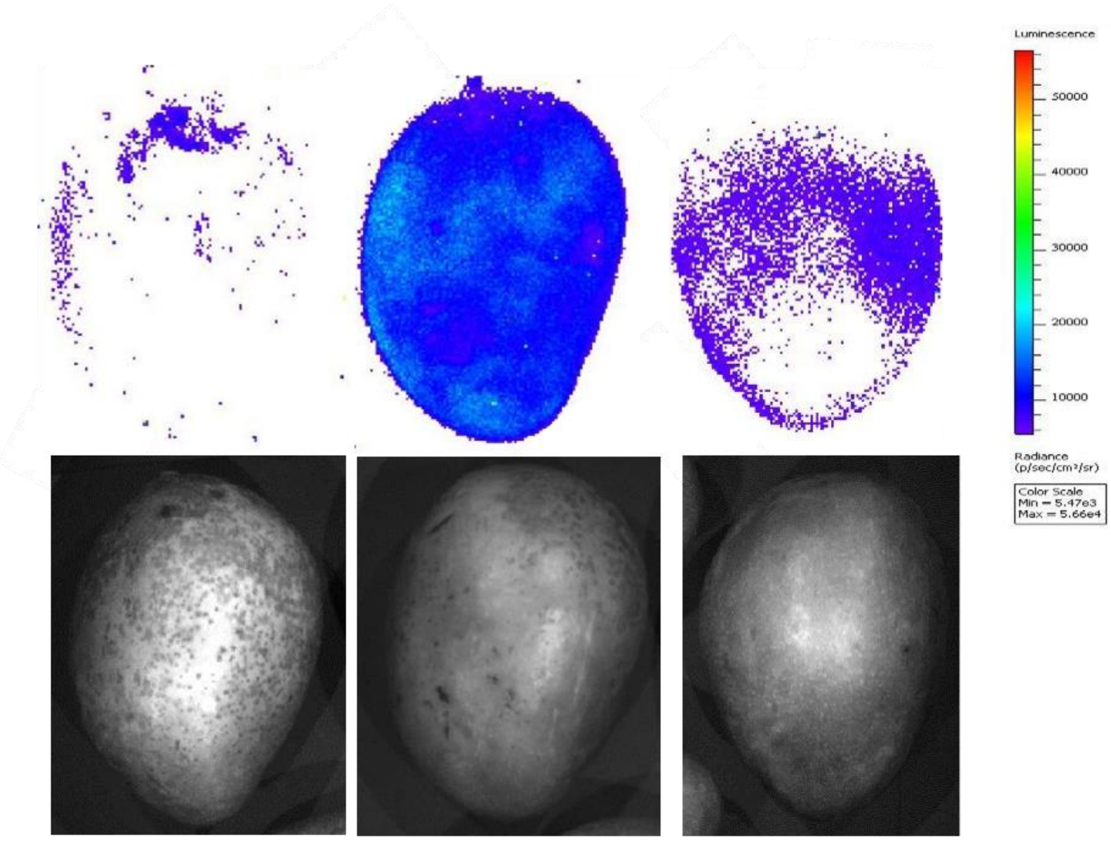

Control AR AR+MA

Supplementary Fig. S2. Lipid peroxidation and luminescence after cold quarantine (19 days at 2°C) of mango fruits of cultivar Keitt in the year 2017. Luminescence over 600 nm indicates lipid peroxidation for artificial ripening (AR: middle upper panel) treatments and combined with modified atmosphere (AR+MA: right upper panel) and Control (left upper panel). Black and white pictures of the same fruits are presented at the lower panel.

Exploring cold quarantine to mango fruit against fruit fly using artificial ripening

Abhinandan S. Patil*, Dalia Maurer*, Oleg Feygenberg & Noam Alkan

Supplementary info S1. Fruit volatiles characterized by GC-MS analysis in the peel of control fruit or in the artificial ripening (AR) treatments or combined with modified atmosphere (MA). Presented are the average and standard deviation of the volatiles in ng/g FW.

R.T.	ng/g FW		Control		AR		AR+MA	
**Aldehyde**			**AVG**	**Std**	**AVG**	**Std**	**AVG**	**Std**
11.202	cis-3-Hexenyl Acetate		**910**	910	**110**	56	**30**	30
2.399	Ethyl Acetate		**0**	0	**48**	48	**0**	0
1.604	Acetaldehyde		**87**	0	**48**	25	**40**	20
3.269	Pentanal	Valeral	**312**	40	**272**	17	**268**	28
5.177	Hexanal		**435**	134	**418**	78	**268**	50
6.52	2-Hexenal		**589**	240	**580**	93	**1092**	490
14.307	Nonanal		**173**	8	**44**	44	**0**	0
**Alcohols**								
1.703	Ethanol		**84**	15	**12**	12	**0**	0
3.077	Ethyl vinyl carbinol		**4**	2	**3**	3	**0**	0
4.479	1-Pentanol	Amyl alcohol	**10**	1	**11**	1	**7**	0
6.615	3-Hexen-1-ol	cis-3-Hexenol	**208**	75	**196**	31	**217**	31
6.974	Isohexanol		**24**	1	**12**	6	**7**	7
**MonoTerpene**								
8.933	1R-α-Pinene		**25**	8	**26**	17	**43**	13
10.736	Myrcene	á-Myrcene	**0**	0	**0**	0	**0**	0
10.752	β-Pinene		**73**	47	**91**	56	**189**	110
11.188	α-Phellandrene		**13**	13	**8**	8	**15**	8
11.369	1R-α-Pinene		**0**	0	**102**	102	**0**	0
11.532	Cyclofenchene	3-Carene	**1432**	568	**1506**	858	**2443**	698
11.607	α-Terpinen		**5**	5	**12**	7	**21**	11
11.826	o-Isopropyltoluene	*p*-Cymene	**9**	9	**18**	10	**31**	12
11.971	Limonene		**63**	39	**87**	50	**149**	69
12.555	β-cis-Ocimene		**0**	0	**0**	0	**3**	3
12.917	Gamma terpinene		**0**	0	**0**	0	**4**	4
13.796	à- Terpinolen	Terpinolen	**5**	5	**11**	6	**20**	10
13.873	à- Terpinolen	Terpinolen	**52**	38	**87**	50	**169**	92
	Terpinolen		**57**	43	**98**	56	**189**	102
18.934	Verbenone		**0**	0	**0**	0	**3**	3
**SesquiTerpene**								
22.605	Copaene		**6**	6	**0**	0	**6**	6
23.94	Caryophyllene	β-Caryophyllen	**895**	337	**282**	124	**1041**	704
24.084	Undecane, 10,10-dimethyl-2,6-bis(methylene)		**8**	8	**0**	0	**12**	12
23.58	α-Gurjunene		**29**	3	**19**	10	**41**	21
24.694	11-Trimethyl-8-methylenebicyclo		**0**	0	**0**	0	**6**	6
24.823	α-Caryophyllene	Humulene	**424**	175	**135**	60	**572**	413
25.191	Longifolene-(V4)		**0**	0	**0**	0	**11**	11
25.672	β-Selinene		**81**	28	**25**	13	**127**	102
25.845	Eremophilene		**0**	0	**0**	0	**8**	8
25.904	α-Selinene		**8**	8	**0**	0	**19**	19

Exploring cold quarantine to mango fruit against fruit fly using artificial ripening

Abhinandan S. Patil*, Dalia Maurer*, Oleg Feygenberg & Noam Alkan

Supplementary info S2. Transcript abbreviations and expression values adapted from Sivankalyani et al. (2016).

Ethyelene biosynthesis										
Acronyms	Transcripts	Seq. Description	KEGG IDs	Harvest (RPKM)	2d_5t (RPKM)	2d_12t (RPKM)	7d_5t1 (RPKM)	7d_12t (RPKM)	14d_5t (RPKM)	14d_12t (RPKM)
metK 2	comp5215_c0_seq1	metk2_elaum ame: s-adenosylmethionine synthase	K00789	232.8365	296.298	75.364	618.186	58.493	419.587	126.8595
speD i	comp20645_c0_seq1	s-adenosylmethionine synthetase	K00789	12.5405	43.4405	3.253	35.1125	5.6145	18.2805	4.1575
speD ii	comp20254_c0_seq1	s-adenosylmethionine synthase 2	K00789	24.623	54.727	4.3225	46.1605	6.3795	19.954	7.565
speD iii	comp20931_c0_seq1	s-adenosylmethionine synthase-like protein	K00789	117.931	169.1275	96.0145	105.2605	50.03	89.048	34.644
ACS i	comp19011_c0_seq1	s-adenosylmethionine decarboxylase proenzyme	K01611	10.254	37.9145	6.6675	35.548	6.782	57.3445	8.257
ACS ii	comp19531_c0_seq1	af512545_1s-adenosylmethionine decarboxylase	K01611	306.303	1061.127	303.2945	570.0195	151.2275	590.202	195.131
ACO	comp19468_c0_seq1	1-aminocyclopropane-1-carboxylate oxidase	K05933	1210.499	2745.828	180.1395	722.8195	38.256	172.469	290.031
Etheylene signal trasduction										
EIN 3 i	comp17311_c0_seq1	ethylene-insensitive 3d	K14514	148.791	368.66	81.611	113.3475	57.556	68.1985	58.752
EIN 3 ii	comp19306_c0_seq1	ethylene-insensitive 3b	K14514	73.023	166.004	37.085	68.2755	30.7595	46.75	38.463
EIN 3 iii	comp19306_c0_seq2	ethylene-insensitive 3b	K14514	100.159	198.252	52.9865	82.4785	43.233	51.3815	47.5815
ERF 1	comp6324_c0_seq1	ethylene-responsive transcription factor	K14516	3.9695	71.5925	2.55	27.4395	1.3875	9.7835	19.125
ERF 2 i	comp14120_c0_seq1	ethylene-responsive transcription factor erf061	K09286	7.4305	80.523	4.1265	26.55	7.27	8.1655	13.998
ERF 2 ii	comp15477_c0_seq1	ethylene-responsive transcription factor wri1	K09285	5.233	11.0085	5.0215	36.7835	9.8875	17.6	22.2545
ERF 2 iii	comp4705_c0_seq1	ethylene responsive element binding protein 2	K09286	6.53	229.788	15.8435	504.83	13.158	424.674	20.447
Sugar metabolism and ethanol formation										
INV i	comp25883_c0_seq1	probable galactinol--sucrose galactosyltransferase 2-like	K06617	2.2145	8.1275	2.486	18.8255	2.0035	24.883	2.878
INV ii	comp18211_c0_seq1	galacturonosyltransferase 13 isoform 1	K13648	5.6765	29.0665	4.9385	22.908	3.1955	23.7395	4.148
INV iii	comp18211_c0_seq2	galacturonosyltransferase 13 isoform 1	K13648	9.3055	42.9095	6.6345	35.737	4.769	31.893	6.2385
INV iv	comp24691_c0_seq1	galactosyltransferase1 isoform partial	K14413	2.051	6.1885	1.6695	10.899	1.307	6.507	1.4705
SS	comp18711_c0_seq1	sucrose synthase	K00695	112.834	198.8495	40.476	129.7985	17.441	227.258	18.018
HK	comp14770_c0_seq1	PREDICTED: fructokinase-2-like	K00847	139.2125	278.427	60.2295	274.2715	42.7905	155.7655	42.9065
pgi	comp16611_c0_seq1	mannose-6-phosphate type i	K01809	10.1175	12.2525	7.65	35.7295	5.541	51.589	6.5505
scrK	comp14770_c0_seq1	PREDICTED: fructokinase-2-like	K00847	139.2125	278.427	60.2295	274.2715	42.7905	155.7655	42.9065
glgC	comp27454_c0_seq1	adp glucose pyrophosphorylase large subunit 1 isoform 1	K00975	1.5885	5.1125	4.7555	5.915	4.8595	12.1385	2.494
pfkA i	comp21147_c0_seq1	6-phosphofructokinase 3-like	K00850	21.0595	130.597	21.2415	149.5415	19.086	155.4485	15.6465
pfkA ii	comp22663_c0_seq1	phosphofructokinase 3 isoform partial	K00850	10.089	59.473	14.4145	74.3625	13.8755	98.3245	13.2885
pfkA iii	comp15819_c0_seq1	6-phosphofructokinase 3	K00850	60.62	241.556	57.6365	184.9955	52.453	89.647	50.1315
pfp i	comp8557_c0_seq1	pyrophosphate--fructose 6-phosphate 1-phosphotransferase subunit beta	K00895	10.304	20.292	5.1025	21.37	6.311	34.8585	3.9255
pfp ii	comp571_c0_seq1	pyrophosphate--fructose 6-phosphate 1-phosphotransferase subunit beta-like	K00895	3.838	7.369	3.041	7.821	2.9635	20.197	2.489
PK i	comp1246_c0_seq1	pyruvate cytosolic isozyme-like	K00873	2.232	8.4655	2.7585	90.4975	2.811	117.7385	1.6465
PK ii	comp26767_c0_seq1	pyruvate cytosolic isozyme-like	K00873	3.2485	8.4305	2.8555	111.0755	4.1185	119.304	1.6865
pckA i	comp12627_c0_seq1	phosphoenolpyruvate carboxykinase	K01610	140.3815	196.8665	94.686	102.4725	68.0885	31.528	68.111
pckA ii	comp7824_c0_seq1	phosphoenolpyruvate carboxylase-related kinase isoform 2	K13412	14.273	20.9005	8.4345	17.631	8.116	4.3555	10.541
PDHA i	comp10913_c0_seq1	pyruvate dehydrogenase e1 component subunit alpha-like	K00161	18.7885	17.3805	21.808	69.1305	33.585	196.7225	32.9505
PDHA ii	comp18860_c0_seq2	pyruvate dehydrogenase e1 alpha	K00161	35.251	30.3	25.1845	56.686	20.4085	107.956	24.662
PDHB	comp18236_c0_seq4	pyruvate dehydrogenase e1 component subunit beta- mitochondrial-like	K00162	42.2555	53.824	21.648	89.9995	18.428	63.1045	24.8025
PDHC	comp20105_c0_seq1	pyruvate decarboxylase	K01568	14.5495	51.0295	48.969	44.4375	59.607	43.43	35.3625
CoA ligase	comp26869_c0_seq1	4-coumarate:coenzyme a ligase	K01904	6.219	7.5795	5.621	43.463	5.3705	8.19	8.1705
ALDH	comp20201_c0_seq1	aldehyde dehydrogenase family 2 member c4-like	K03627	29.484	225.73	53.733	262.5525	57.054	251.0615	32.1415
ADH5	comp42190_c0_seq1	alcohol dehydrogenase-like 7-like	K00121	0.5595	0.2055	1.146	1.1545	1.4245	3.9675	0.2605
